# Novel Strategy for the Management of Cervical Multicystic Diseases

**DOI:** 10.1245/s10434-022-13033-7

**Published:** 2023-03-15

**Authors:** Ai Yoshino, Eiji Kobayashi, Takahiro Tsuboyama, Hideyuki Fukui, Noriyuki Tomiyama, Kazuaki Sato, Eiichi Morii, Eiji Nakatani, Naoko Komura, Ikuko Sawada, Yusuke Tanaka, Kensuke Hori, Akihiko Yoshimura, Ryoko Takahashi, Tadashi Iwamiya, Tsuyoshi Hisa, Sadako Nishimura, Toshihiro Kitai, Hiromi Yokota, Mariko Shindo, Hiromi Miyata, Namiko Hashimoto, Kanako Sakiyama, Hazuki Abe, Yutaka Ueda, Tadashi Kimura

**Affiliations:** 1 Department of Obstetrics and Gynecology, Osaka University Graduate School of Medicine, Osaka, Japan; 2 Department of Radiology, Osaka University Graduate School of Medicine, Osaka, Japan; 3 Department of Pathology, Osaka University Graduate School of Medicine, Osaka, Japan; 4 Graduate School of Public Health (Medical Statistics), Shizuoka Graduate University of Public Health, Shizuoka, Japan; 5 Department of Obstetrics and Gynecology, Kaizuka City Hospital, Osaka, Japan; 6 Department of Obstetrics and Gynecology, Itami City Hospital, Itami, Hyogo Japan; 7 Department of Obstetrics and Gynecology, Osaka Rousai Hospital, Osaka, Japan; 8 Department of Obstetrics and Gynecology, Kansai Rousai Hospital, Amagasaki, Hyogo Japan; 9 Department of Obstetrics and Gynecology, Bell Land General Hospital, Osaka, Japan; 10 Department of Obstetrics and Gynecology, Toyonaka Municipal Hospital, Osaka, Japan; 11 Department of Obstetrics and Gynecology, Osaka General Medical Center, Osaka, Japan; 12 Department of Obstetrics and Gynecology, Osaka International Cancer Institute, Osaka, Japan; 13 Department of Obstetrics and Gynecology, Sumitomo Hospital, Osaka, Japan; 14 Department of Obstetrics and Gynecology, Hyogo Prefectural Nishinomiya Hospital Hyogo, Nishinomiya, Japan; 15 Department of Obstetrics and Gynecology, Suita Municipal Hospital, Osaka, Japan; 16 Department of Obstetrics and Gynecology, Hannan Chuo Hospital, Osaka, Japan; 17 Department of Obstetrics and Gynecology, Ashiya Municipal Hospital, Ashiya, Hyogo Japan; 18 Department of Obstetrics and Gynecology, Nippon Life Hospital, Osaka, Japan

## Abstract

**Purpose:**

To investigate the clinical practices of diagnosing multicystic cervical lesions as a means to develop a more appropriate diagnostic algorithm for gastric-type adenocarcinoma (GAS) and its precursors.

**Methods:**

Clinical information for 159 surgically treated patients for multicystic disease of the uterine cervix was collected from 15 hospitals. We performed a central review of the MRI and pathological findings. The MRI findings were categorized into four types including two newly proposed imaging features based on the morphology and distribution of cysts, and the diagnosis accuracy was assessed. Among the four MRI types, types 1 and 2 were categorized as benign lesions that included LEGH; type 3 were precancerous lesions (with an assumption of atypical LEGH); and type 4 were malignant lesions.

**Results:**

The central pathological review identified 56 cases of LEGH, seven with GAS, four with another form of carcinoma, and 92 with benign disease. In clinical practice, over-diagnosis of malignancy (suspicion of MDA) occurred for 12/19 cases (63.2%) and under-diagnosis of malignancy occurred for 4/11 (36%). Among the 118 patients who had a preoperative MRI and underwent a hysterectomy, type 3 or 4 MRI findings in conjunction with abnormal cytology were positively indicative of premalignancy or malignancy, with a sensitivity and specificity of 61.1% and 96.7%, respectively.

**Conclusions:**

Although the correct preoperative diagnosis of cervical cancer with a multicystic lesion is challenging, the combination of cytology and MRI findings creates a more appropriate diagnostic algorithm that significantly improves the diagnostic accuracy for differentiating benign disease from premalignancy and malignancy.

**Supplementary Information:**

The online version contains supplementary material available at 10.1245/s10434-022-13033-7.

Lobular endocervical glandular hyperplasia (LEGH) was first described as a benign lobular proliferation of small glands lined by mucin-producing endocervical epithelial cells without nuclear atypia nor stromal invasion.^1^ Subsequent studies demonstrated that a proportion of LEGH might be a precursor of gastric-type adenocarcinoma (GAS) and minimal deviation adenocarcinoma (MDA), because there is common gastric differentiation and frequent coexistence of these two conditions.^2–8^

GAS and MDA are associated with a significantly lower 5-year overall survival rate compared with that of the usual-type endocervical adenocarcinoma (UEA).^9–14^ The cause of the poor prognosis for GAS is unclear; however, it may be attributed to a highly infiltrating pattern of growth, retardation of timely diagnosis, and its refractoriness to chemotherapy and radiotherapy.^10–15^

Cervical cystic lesions are often difficult to distinguish from malignant lesions in routine clinical practice. Due to the recent spread of transvaginal ultrasound, multicystic lesions are increasingly found by chance, which leads to one of the causes of management confusion in clinical practice. In the past, the presence of cervical cysts led to an over-diagnosis and many cases of unnecessary radical hysterectomy.^16^

Although a management protocol for patients with cervical multilocular lesions has been proposed, few of the underlying studies were based on a sufficiently large case series. A preoperative differential diagnosis between MDA, LEGH, and other cervical multilocular lesions is still problematic in primary clinical practice. In this study, we have retrospectively reviewed a large number of surgically treated cases of multicystic cervical lesions with a suspicion of GAS, MDA, or LEGH. We looked at their imaging and central pathological reviews to investigate the current status of their management, and we researched to develop an algorithm for their improved diagnosis and treatment.

## Methods

### Patient Selection

Our study was approved by the Institutional Review Boards of the Osaka University Graduate School of Medicine and the relevant Boards of all 15 participating hospitals. Eligible patients were those who underwent hysterectomy or conization at the participating hospitals between 2010 and 2019 due to an indication of a multicystic cervical lesion. Clinical records included a Pap smear, a cervical biopsy specimen with hematoxylin-eosin (H&E) staining, MRI, and H&E-stained sections of the surgically resected uterus or cone biopsies. The diagnoses, made by each participating hospital, were collected by the Osaka University Department of Obstetrics and Gynecology. Additional patient data, including age, gravidity, past medical and obstetric history, BMI, tumor markers, surgical procedure details, adjuvant therapy, and the results of follow-ups, were retrospectively collected and reviewed.

### Pathologic Evaluation

The H&E-stained sections of hysterectomy or conization specimens were re-evaluated by a central pathological review (CPR) consisting of two board-certified pathologists specializing in gynecological pathology and oncology, without access to the case clinical information.

The first pathologist reviewed all of the slides. A second pathologist was consulted only when the first central pathological diagnosis was subsequently found to be discordant with the initial diagnosis made at the participating hospital.

The final diagnosis was made based on the 2020 WHO classification system.^17^ GAS was defined as an invasive adenocarcinoma with abundant pale eosinophilic or clear cytoplasm and distinct cell borders, and destructive invasion and moderate to severe nuclear atypia. MDA was categorized as an extremely well-differentiated variant of GAS.^18^ LEGH was defined as cyst-like large central glands surrounded by a lobular proliferation of small- to medium-sized glands containing tall columnar mucinous epithelium, sometimes with eosinophilic, granular cytoplasm, and bland, basally located nuclei. Adenocarcinoma in situ (AIS) and atypical LEGH were defined as follows: (i) AIS is characterized by mucin-producing cells as seen in cases of GAS. The proliferation involves pre-existing normal endocervical glands and lacks haphazard gland crowding or confluence, but intraglandular complexity can occur; (ii) atypical LEGH was defined as tumors with the architecture of prototypical LEGH preserved, characterized by epithelial infoldings, tufts, or papillae, as well as by nuclear hyperchromasia, enlargement, distinct nucleoli, apoptotic bodies, and occasional apical mitoses, but do not suffice as carcinoma. In this limited study, we did not perform additional immunohistochemical diagnostic analysis.

### Image Analysis

We used MRI to categorize malignant potential by classifying the morphology and distribution of cysts, as follows:

1. The location of the lesion was classified into four parts (upper part, lower part, middle part, and the entire cervix).
2. The cyst and solid component patterns were categorized into four patterns as well:
  ① “Microcystic pattern”—only small cysts ≤ 3 mm were present,
  ② “Macrocystic pattern”—only cysts > 3 mm were present,
  ③ “Mixed pattern”—admixed microcysts and macrocysts (Supplemental Table 1),
  ④ “Solid and cystic pattern”—lesion comprised multiple cysts and solid components.

A ‘solid component’ was defined as a nodular or infiltrative solid tumor area, with diffusion restriction indicated by hyper-intensity on the diffusion-weighted imaging (DWI) and hypo-intensity on the apparent diffusion coefficient (ADC) map, relative to the uterine myometrium, suggesting possible malignancy. A solid-like hyper-intensity area seen at the center of a cosmos pattern was not regarded to be a solid component.

**Fig. 1:**
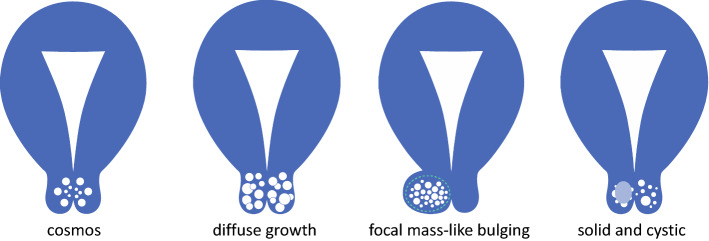
Definitions of MRI morphological patterns in this study. “Cosmos sign” was defined as an aggregation of small cystic components in the center of the lesion, occasionally seen as a solid-like less hyper-intense area on T2WI, surrounded by many larger cysts. “Diffuse growth” was defined as microcysts distributed diffusely across the entire cervix and deeper than half of the cervical wall. “Focal mass-like bulging pattern” was defined as a mass-like lesion composed of numerous cysts forming a focal outward contour bulge of the cervical wall unilaterally. “Sold and cystic pattern” lesions comprised multiple cysts and a solid component

**Fig. 2:**
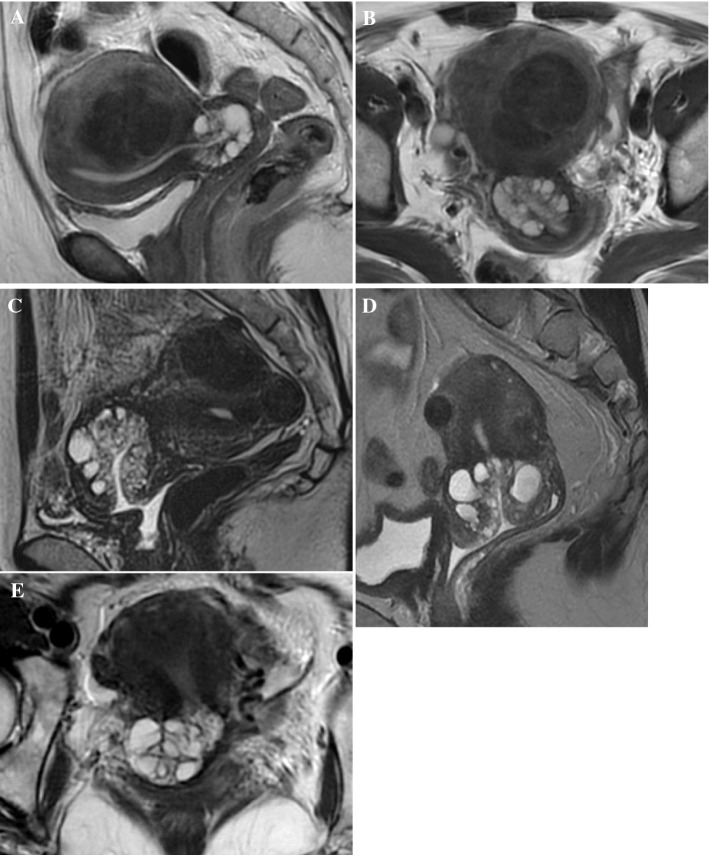
Typical MR Images. T2-weighed MR images of “cosmos” (**A**, **B**), and novel morphological categories of cystic components, “diffuse growth” (**C**, **D**) and “focal mass-like bulging” (**E**). **A** and **B**: A case of LEGH showing “cosmos”. There are multiple microcysts surrounded by macrocysts within the tumor at sagittal and axial T2-weighted image. **C** and **D**: Sagittal T2-WI showing “diffuse growth” observed in GAS cases. Microcysts exceeding 1/2 of the cervical stroma extend from external to internal cervical os. **E**: A case of atypical LEGH showing “focal mass-like bulging”. A cervical lesion is shown as an eccentric protrusion of round, mass-like multifocal cysts on axial T2-WI

(3) The following three special morphologies of the cystic components were evaluated: ① “cosmos sign”, an aggregation of small cystic components in the center of the lesions, occasionally seen as a solid-like less hyper-intense area on a T2-weighted image (T2WI) surrounded by many larger cysts,
  ② “diffuse growth”, microcysts distributed diffusely across the entire cervix and involving deeper than half of the cervical wall,
  ③ “focal mass-like bulging pattern”, a mass-like lesion composed of numerous cysts forming a focal outward contour bulge of the cervical wall unilaterally (illustrated in Figs. 1 and 2, and Supplemental Table 1).

Two gynecologic radiologists independently assessed the above radiologic features of the tumor, without knowing any of the patients’ original diagnosis or clinical information. Based on the morphology and distribution of the cysts, each radiologist made a radiological diagnosis according to the diagnostic imaging algorithm described above. Lesions were classified into four types based on the above-described cyst and solid component patterns. Types 1 to 3 comprised only a multicystic component. Type 1, “lesion confined to the lower portion of the cervix,” “macrocystic pattern,” or “mixed pattern without cosmos sign,” were regarded as suspicious of miscellaneous benign glandular hyperplasia, including a nabothian cyst (NC); type 2, “cosmos pattern or microcystic pattern without diffuse growth and focal mass-like bulging” were regarded as suspicious of possibly LEGH; type 3, a mixed or microcystic pattern exhibiting “diffuse growth,” or “focal mass-like bulging” were regarded as suspicious of premalignancy (assumption of atypical LEGH) or possible malignancy; type 4, “sold and cystic pattern,” were regarded as suspicious of malignancy (Table 1).

**Table 1:** Classification of MRI findings

Type	Suspected disease	Radiological findings
1	Benign (non LEGH)	Lower portion
		Macrocystic lesion
		Mixed lesion
		Cosmos sign –
		Solid lesion –
2	Benign (possible LEGH)	Mixed lesion with cosmos sign
		Microcystic lesion
		Diffuse growth –
		Focal mass-like bulging –
		Solid portion –
3	Malignant (possible premalignant or malignant)	Mixed or microcystic lesion
		Diffuse growth +
		Focal mass-like bulging +
		Solid portion –
4	Malignant (definite malignant)	Solid portion

*LEGH* lobular endocervical glandular hyperplasia

We investigated the diagnostic value of various clinical parameters, including MRI and cytology, and evaluated their usefulness for diagnostic accuracy regarding multicystic diseases of the cervix using a combination of these parameters.

### Statistical Analysis

Continuous variables were summarized as mean ± standard deviation and categorical variables as frequency and percentage. The Cochran-Armitage trend test was used to ascertain whether malignant or atypical LEGH increases in the order of type 4 morphology of MRI. The McNemar’s test was used to check whether cytology results were consistent with pathology results. To assess the associations between MRI findings and histopathological diagnosis, Fisher’s exact test and the Freeman-Halton test were used. Statistical significance was determined when the *p*-value was less than 0.05. JMP software, version 16.0, (SAS Institute, Cary, NC, USA) and SAS version 9.4 (SAS Institute Inc., Cary, NC, USA) were used for the statistical analysis.

## Results

A total of 180 patients, from 15 hospitals, were initially registered for this study. We subsequently excluded 21 patients with cervical proliferative glandular lesions only incidentally detected as part of surgery performed for some other benign disease. Finally, 159 cases were included in the analysis. Their mean age was 52 years (range 31–80). Among the 159 cases of a cervical multicystic disease diagnosed at the 15 participating hospitals, the preoperative diagnosis was: 19 cases of suspected cancer, 21 cases of suspected benign disease, and 119 cases that were ‘suspicious of LEGH’ or ‘suspicious of LEGH—but MDA cancer cannot be ruled out’ (Table 2). Clinical over-diagnosis of malignancy (suspicion of MDA) occurred for 12/19 cases (63.2%) and under-diagnosis of malignancy occurred for 4/11 (36%).

**Table 2:** Patient characteristics

Clinical diagnosis	No. Cases	Age Mean ± SD	Watery discharge n (%)	Irregular bleeding n (%)	Abnormal cytology (Gl. atypia) n (%)	Incidence of malignancy after CPR n (%)
S/O MDA or Ca	19	46.0±5.0	6 (33.3%)	6 (33.3%)	9 (50.0%)	7 (36.8%)
S/O LEGH	119	58.0±11.0	31 (26.3%)	21 (17.8%)	14 (12.7%)	3 (2.5%)
NC, TC, etc.	21	56.0±10.0	6 (31.6%)	5 (26.3%)	1 (5.0%)	1 (4.8%)
Total	159	52.0±3.0	43 (27.4%)	33 (21.3%)	24 (16.0%)	11 (6.9%)

*Gl*. atypia, glandular atypia; *S/O MDA* or *Ca*, suspicious of minimal deviation adenocarcinoma or carcinoa; *S/O LEGH*, suspicious of lobular endocervical glandular hyperplasia; *NC*, nabothian cyst; *TC*, tunnel cluster

### Results of Central Pathological Review (CPR)

Table 3 shows the differences between the pathological diagnosis at the participating hospitals and the results of our central pathological review (CPR). The pathological diagnoses made at the participating hospitals were 20 cancer cases (14 GAS, including MDA, and 6 other types of carcinomas), 84 miscellaneous benign glandular diseases, and 55 LEGH (including atypical LEGH). Of the 55 LEGH patients, 45 were categorized with LEGH and 10 with other benign glandular proliferative diseases. Of 84 cases of miscellaneous benign glandular disease other than LEGH, the CPR diagnosis was mostly consistent with the original diagnosis (80 benign diseases and 4 LEGH). Of note, of 20 cases locally diagnosed with malignant disease (before CPR), only 11 cases (55.0%) were confirmed to be malignant disease, and 9 (45.0%) were diagnosed by CPR as being benign or as atypical LEGH.

**Table 3:**
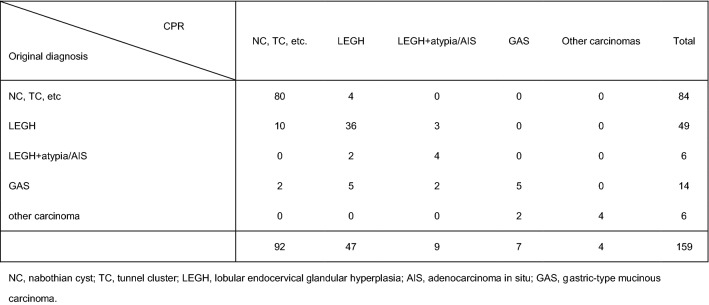
Original diagnosis and CPR results

Of the 14 cases initially diagnosed as MDA or GAS, CPR revealed that only five cases (35.7%) were GAS or MDA. Two were atypical LEGH, five were pure LEGH, and two were miscellaneous benign glandular diseases. (Table 3 and Supplemental Table 2). Finally, CPR revealed 56 cases of LEGH (including nine atypical LEGH), seven with GAS including MDA, four with other types of invasive carcinoma, and 92 cases of miscellaneous benign glandular proliferative disease (Supplemental Table 2).

Pathological diagnosis was made by analysis of the hysterectomy specimen in 125 cases, and from the conization specimen in 34 cases. The patients’ characteristics are summarized in Table 4.

**Table 4:** Clinocopathologic summary of patients

Characteristics	Review diagnosis				
	NC, TC, etc.	LEGH	LEGH+atypia/AIS	GAS	Other carcinoma
	(n=92)	(n=47)	(n=9)	(n=7)	(n=4)
Mean age (range), y	47 (33-80)	49.5 (31-68)	49.5 (43-76)	47 (34-53)	48 (32-49)
Symptoms					
Watery discharge	20 (21.7%)	17 (36.2%)	4 (44.4%)	3 (42.9%)	0
Vaginal bleeding	20 (21.7%)	9 (19.1%)	1 (11.1%)	1 (14.3%)	1 (25.0%)
Other	17 (18.4%)	5 (10.6%)	2 (22.2%)	0	1 (25.0%)
None	35 (38.0%)	15 (31.9%)	3 (33.3%)	3 (42.9%)	2 (50%)
Cytology					
NILM/ASC/SIL	80 (90.9%)	38 (86.4%)	3 (33.3%)	3 (50%)	1 (25.0%)
AGC	7 (8.0%)	6 (13.6%)	3 (33.3%)	1 (16.7%)	2 (50%)
AIS	0	0	1 (11.1%)	0	0
Adenocarcinoma	1 (1.1%)*	0	2 (22.2%)	2 (33.3%)	1 (25.0%)
N/A	4	3	0	1	0
Cervical biopsy	n=40	n=25	n=6	n=4	n=4
No malignancy	30 (75.0%)	21 (84.0%)	3 (50%)	2 (50%)	0
Hyperplasia, atypical glands	2 (5.0%)	2 (4.0%)	0	0	0
NC, TC	1 (2.5%)		0	0	0
LEGH	0	2 (4.0%)	1 (16.7%)	0	0
CIN	6 (15.0%)	0	1 (16.7%)	0	1 (25.0%)
AIS	0	0	1 (16.7%)	0	1 (25.0%)
Adenocarcinoma	1 (2.5%)*	0	0	2 (50%)	2 (50%)
Conization	n=33	n=13	n=2	n=1	n=2
No malignancy	5 (15.2%)	2 (15.4%)	0	0	0
Hyperplasia, atypical glands	5 (15.2%)	3 (23.1%)	0	0	0
NC,TC	22 (66.7%)	1 (7.7%)	1 (50%)		
LEGH	0	6 (46.2%)	0	0	0
CIN, SCC	1 (3.0%)	1 (7.7%)	0	0	1 (50%)
AIS	0	0	1 (50%)	0	0
GAS	0	0	0	1 (100%)	0
Adenocarcinoma	0	0	0	0	1 (50%)
FIGO stage					
IB1				4 (57.1%)	4 (100%)
IB2				2 (28.6%)	0
IIA2				1 (14.3%)	0
Mode of surgery					
Conization, tumorectomy	30 (32.6%)	4 (8.5%)	0	0	0
SH	54 (58.7%)	31 (66.0%)	6 (66.6%)	3 (42.9%)	0
Modified RH	7 (7.6%)	10 (21.3%)	1 (11.1%)	1 (14.3%)	0
RH	1 (1.1%)	2 (4.3%)	2 (22.2%)	3 (42.9%)	4 (100%)
Outcome					
NED				7 (100%)	3 (75.0%)
Recurrence				0	1 (25.0%)
DOD				0	0

This table consists of the patients whose relevant data are available

*A case concurred with ovarian cancer

*NC* nabothian cyst, *TC* tunnel cluster, *LEGH* lobular endocervical glandular hyperplasia, *AIS* adenocarcinoma in situ, *GAS* gastric-type mucinous carcinoma, *CIN* cervical intra-epithelial neoplasia, *SCC* squamous cell carcinoma, *FIGO* International Federation of Gynecology and Obstetrics, *SH* simple hysterectomy, *RH* radical hysterectomy, *NED* no evidence of disease, *DOD* died of disease, *NA* not available

Although there were 31 radical or modified radical hysterectomies performed at the participating hospitals, only eight of the 31 cases (25.8%) were diagnosed as actual cervical cancer by CPR.

### Diagnosis of Lobular Endocervical Glandular Hyperplasia (Including Atypical LEGH)

Of the 56 LEGH cases (after CPR), 47 were LEGH alone, and 9 were associated with cellular atypia (atypical LEGH) (Supplemental Table 4). Four, 37, 11, and four received conization, simple hysterectomies, modified radical hysterectomies, and radical hysterectomies with pelvic lymph node dissection, respectively (Table 4). Cytologically, only six (13.6%) of 44 pure LEGH showed atypical glandular cells (AGC), whereas six (66.7%) of nine LEGH with atypia showed AGC, AIS, or adenocarcinoma. Among the 31 patients who underwent punch biopsy, only three (9.7%) were diagnosed as having LEGH. Of six LEGH with atypia whose punch or cone biopsy results were available, only one (16.7%) was a premalignant lesion. Of 15 patients who underwent diagnostic conization, six (40%) revealed LEGH. Of two LEGH with atypia that underwent cone biopsy, one (50.0%) was diagnosed as AIS.

### Diagnosis of Malignant Diseases

The clinicopathological data of 11 patients with malignant disease (after CPR) are summarized in Supplemental Table 3. Of these 11 patients, seven were diagnosed with GAS, three with UEA, and one with squamous cell carcinoma (SCC). Four out of the seven GAS and two out of the four other types of carcinomas were associated with LEGH. In six out of seven available GAS patient cytological reports, three (50.0%) had abnormal cervical cytology (one AGC, and two adenocarcinomas). Of the four GAS cases where results of punch biopsy were available, a malignant lesion was detected in two patients (50.0%). The one case with GAS who underwent conization was diagnosed as having GAS (1/1, 100%). In contrast, all patients with other types of carcinomas were detected as malignancy by cytological evaluation and punch biopsy or conization. Three patients with GAS received radical hysterectomies and pelvic lymph node dissections, and four patients received a simple or modified radical hysterectomy due to a suspicion of LEGH and were diagnosed incidentally post-surgery via the resected uterus, whereas all patients with other types of carcinomas received radical hysterectomies and pelvic lymph node dissection. Postoperatively, four patients received radiotherapy and one patient received chemotherapy. During the median follow-up period of 50 months (ranging from 1 to 62 months), one patient with stage IB1 UEA developed a recurrence at the vaginal stump 15 months after the initial surgery and is still alive with the disease.

### MRI Findings and the Possibility of Using Them for Preoperative Diagnosis

Of the 159 enrolled patients, 118 who had both MRI data and pathological diagnosis by hysterectomy specimen were subjected to our algorithm analysis. There were 99 benign diseases (40 LEGH cases; 59 other benign proliferating disease cases), nine premalignant diseases (seven atypical LEGH cases and two cases of LEGH with AIS), and ten malignant diseases (GAS, 7 cases; other adenocarcinomas, 3 cases). The category of MRI findings and final pathological diagnoses are presented in Table 5 and Supplemental Table 5.

**Table 5:** Correlation between MRI findings and pathological diagnosis

MR findings	NC, TC, etc.	LEGH	Precursors	Malignancy	Total
	(n=59)	(n=40)	(n=9)	(n=10)	
Type 1	33 (55.9%)	1 (2.5%)	1 (11.1%)	0	35
Type 2	26 (44.1%)	22 (55%)	1 (11.1%)	0	49
Type 3	0	17 (42.5%)	7 (77.8%)	6 (60%)	30
Type 4	0	0	0	4 (40%)	4

*NC* nabothian cyst, *TC* tunnel cluster, *LEGH* lobular endocervical glandular hyperplasia

### Malignant and Premalignant Diseases

Of the ten cases with malignant disease, four (40%) showed type 4 morphology, and six (60%) showed type 3. Of nine LEGH with atypia, seven (77.8%), one (11.1%), and one (11.1%) showed type 3, type 2, and type 1 morphology, respectively. The malignancy or atypical LEGH were observed 2.9% (1/35), 2.0% (1/49), 43.3% (13/30), and 100% (4/4) in types 1 to 4, respectively (*p* < 0.0001 for trend).

When both type 3 or 4 MRI morphologies and abnormal cytology were shown, the sensitivity, specificity, PPV, NPV, and OR for differentiating malignancy or premalignancy were 61.1%, 96.7%, 73.3%, 94.4%, and 46.357, respectively. The *P* value of the McNemar analysis was 0.366, which means that the findings of MRI morphology and cytology results consistently correlated with benign and malignant pathology results.

## Discussion

Our study reveals that an accurate preoperative diagnosis of multicystic cervical lesions is still quite difficult in primary clinical practices. The strong evidence for this is the high discordance in the differential diagnosis between MDA and LEGH among pathologists and radiologists.

In the current study, 57.1% (4/7) of the GAS were not detected as being a malignant lesion by preoperative pathological assessment and were only incidentally found after hysterectomy due to a suspicion of LEGH. Most importantly, 67.4% (62/92) of the miscellaneous benign glandular proliferative disease cases underwent unnecessary hysterectomy due to the suspicion of a LEGH-related disease.

In regards to preoperative pathological diagnosis, a previous study has described the low detection rate of MDA or GAS by cytology as only 32.7%.^19^ In the current study, a cervical Pap smear revealed precancerous or cancerous cells in 42.9% (3/7) of the GAS, which is consistent with the previous report. A superficial punch biopsy is of little value in the diagnosis of MDA because MDA is frequently located in the upper endocervical canal, and atypical cells of MDA likely existed deep in the cervical stroma. Li et al. reported that the detection rate of MDA by all biopsies, including conization, was 50.7%.^19^ In this study, 50% (2/4) of GAS with stage IB1 and IIA2 disease was diagnosed as adenocarcinoma by punch biopsy. Of LEGH with atypia, 16.6% (1/6) was detected as a premalignant lesion by biopsy. It is supposed that the malignant cells of GAS, or LEGH with atypia, with normal cytology, are likely to locate near the internal os and deep in the cervical stroma, making it difficult to obtain parts of the malignant lesion by small biopsy.

Of the 31 patients with LEGH, including LEGH with atypia, who underwent biopsy, only four were properly diagnosed. Therefore, punch biopsy has a very limited value for the diagnosis of LEGH. Cone biopsy was reported to be an effective option for the diagnosis of MDA and LEGH in several studies. In the current study, 46.2% (6/13) of LEGH, 50% (1/2) of LEGH with atypia, and 100% (1/1) GAS with stage IB2 disease were diagnosed by conization. This result suggests that diagnostic conization may provide more useful information compared with the punch biopsy; however, making a definitive diagnosis of LEGH by conization is difficult and there is a risk of overlooking focal MDA coexisting with LEGH.

MRI is widely considered to be useful for the differential diagnosis of multicystic cervical lesions. Takatsu et al. reported that a solid mass often associated with various sized cystic structures suggests MDA and that multiple large cysts and an inner solid-like part composed of aggregated small cysts, the so-called “cosmos sign,” suggests LEGH, and a coarse cystic pattern with a clear margin is suggestive of NC.^16^ However, these diseases frequently show overlapping MRI findings. In our cases, 60% (6/10) of the malignancies and 40.7% (24/59) of the other benign cystic diseases displayed the MRI cosmos pattern, resulting in their misdiagnosis as LEGH, based on previously reported criteria.

As the diagnostic power of biopsy and conventional MRI diagnosis is limited, we proposed a novel category of MRI findings and analyzed its validity. Then, the MRI findings were classified into four types to improve the predictive accuracy of multicystic cervical lesions. All 10 malignant disease cases and 77.8% (7/9) of premalignant disease cases were classified into type 3 or 4 morphology categories. Lesions showing type 4, the mixture of a solid component with diffusion restriction and a cystic component, were highly suspicious of malignancy, with a rate of 100% (4/4).

The presence of a solid component with a diffusion restriction within the lesion is an important finding suggesting malignancy, as described in previous reports.^9,20,21^ When MRI showed a type 4 morphology along with cellular atypia, the case was diagnosed as a malignancy (correct ratio: 3/3, 100%).

The clinical argument surrounds the management of multicystic lesions without a solid component, as reported previously, which corresponds to type 3 in our MRI category.^22^ Few reports focus on the MRI findings of MDA comprising only a cystic component. Omori et al. reported that AIS was identified in postmenopausal women showing a “raspberry-type” lesion with many tiny, closely aggregated cysts. However, in our study, the "raspberry pattern," which corresponds to the microcystic pattern, using our criteria, was not associated with the risk of malignancy.^23^ Other useful indicators for differentiating MDA comprising only a cystic component from benign multicystic diseases are unknown. Type 3, exhibiting “diffuse growth” or “focal mass-like bulging,” is a newly proposed imaging feature in our criteria. In our cases, 60% (6/10) of the malignant lesions and 77.8% (7/9) of the premalignant lesions showed a type 3 MRI morphology**.** Lesions with “diffuse growth” or “focal mass-like bulging” were of significantly higher risk of malignancy or premalignancy (43.3%) compared with those with cosmos patterns without these features.

### Combination of Cervical Cytology and MRI Findings

Cases with type 3 or 4 MRI findings and abnormal cytology were highly suggested to be malignancy or premalignancy, with the sensitivity, specificity, PPV, and NPV for differentiating malignancy or premalignancy being 61.1%, 96.7%, 73.3%, and 94.4%, respectively. Since a false negative on cytological evaluation may occur, type 3 and 4 on MRI may justify immediate biopsy or conization and hysterectomy, regardless of the results of cytology.

Compared with previous reports, the diagnostic accuracy of a management protocol for cervical multicystic lesions by Takatsu et al*.* for suspected malignancy and suspected LEGH was 66.7 to 70% and 69.2 to 90%, respectively.^16,24,25^ In contrast to our series, all the malignant cases in their series showed either a solid or an invasive pattern on MRI, or malignancy by cytology and biopsy, and none were under-diagnosed as LEGH. Therefore, it is difficult to compare the diagnostic accuracy of our protocol with that of previous reports.

Occult cervical cancer, i.e., unsuspected invasive cancer diagnosed by conization or hysterectomy, is reported in recent large studies to have an incidence of 0.31–1.6%.^26,27^ Preoperative diagnosis may not be possible even with common cervical cancer, making it even more challenging with cervical cancer exhibiting multicystic lesions.

From previous reports and our series, the preoperative diagnosis of cervical cystic lesions is still problematic; however, the under-diagnosis of malignant lesions (including pre-invasive diseases) is much lower using our diagnostic criteria.

There are several limitations to our study. First, we retrospectively analyzed only those patients who underwent surgery, which may cause a selection bias. Second, the number of malignant disease patients is limited because of the rarity of GAS and adenocarcinoma associated with LEGH. However, previous reports included only a small number of GAS or MDA cases and, to our knowledge, our study has the largest number of multicystic cervical diseases which were histologically confirmed from surgically resected specimens. Moreover, we included mostly GAS composed predominantly of those with a cystic component on MRI and proposed the novel radiological classification to improve the differential diagnosis accuracy of malignancy comprising only cystic components and premalignancy. Third, the pathological evaluation was performed primarily based on morphology in our study and we could not perform immunohistochemical analysis, including HIK1083 staining. If immunohistochemical analysis had been available, more accurate pathological diagnosis might have been made.

In conclusion, multicystic lesions with type 3 or 4 MRI morphologies were at high risk of premalignancy or malignancy. When the MRI showed a type 3 or 4 morphology in association with at least AGC in the Pap smear, active management, such as biopsy or conization, or hysterectomy, may be reasonable to address the possibility of premalignancy or malignancy. These diagnostic criteria should be evaluated in future prospective studies.

## Supplementary Information

Below is the link to the electronic supplementary material.

Supplementary file1 (DOCX 26 KB)
