# Supplementary material for: Novel Strategy for the Management of Cervical Multicystic Diseases
Source: Ann Surg Oncol. 2023 Mar 15;30(5):2964–73. doi: 10.1245/s10434-022-13033-7 (PMC10085883; doi:10.1245/s10434-022-13033-7)
Supplement: Supplementary file 1 — Supplementary file1 (DOCX 26 KB) [file 10434_2022_13033_MOESM1_ESM.docx]

**Supple Tables**

| **Supple Table 1. Definitions of MRI findings** | | |  |
| --- | --- | --- | --- |
|  | Definition | Details of definition | |
| Solid part |  | including solid component | |
| Cyst size | microcyst | 3 mm > | |
|  | macrocyst | 3 mm≦ | |
| Location of the cyst | lower | Located in the external cervical os and disconnected from the internal os | |
|  | upper | Located in the internal cervical os and disconnected from the external os | |
|  | entire | Located in the entire cervix and connected to both the internal and external cervical os | |
|  | middle | Located in the cervix and disconnected from both the internal and external cervical os | |
| Type of structure | cosmos | The macrocysts are arranged around the microcysts. | |
|  | diffuse growth | The area of microcysts exceeds 1/2 of the cervical stroma and extends from the external to the internal cervical os | |
|  | focal mass-like bulging | Eccentric protrusion of round, mass-like multifocal cyst. Almost no involvement of the contralateral wall. | |

| **Supple Table2. Histological diagnosis by CPR** | |
| --- | --- |
|  | **No. Cases** |
| LEGH | Total=56 |
| LEGH alone | 47 |
| LEGH+atypia | 9 |
| Carcinoma | Total=11 |
| GAS+LEGH | 4 |
| GAS | 3 |
| UEA | 2 |
| UEA+LEGH | 1 |
| SCC+LEGH | 1 |
| Benign glandular lesions | Total=92 |
| NC, TC | 83 |
| Nonspecific gland proliferation | 9 |
| LEGH, lobular endocervical glandular hyperplasia; GAS, gastric-type mucinous carcinoma; SCC, squamous cell carcinoma; UEA, endocervical adenocarcinoma, usual type; NC, nabothian cyst; TC, tunnel cluster. | |

| **Supple Table3. Summary of 11 patients with malignant disease** | | | | |  |  |  |  |  |
| --- | --- | --- | --- | --- | --- | --- | --- | --- | --- |
| Case  No | **Age** | **MRI  findings** | **MRI type** | **Cytology** | **Cervical biopsy** | **Conization** | **Mode of  surgery** | **Postoperative diagnosis** | **FIGO stage** |
| 1 | 49 | solid and cystic | 4 | AGC | adenocarcinoma | N/A | RH | UEA | IB1 |
| 2 | 47 | mixed/diffuse | 3 | NILM | No malignancy | N/A | SH | GAS | IB1 |
| 3 | 47 | solid and cystic | 4 | adenocarcinoma | adenocarcinoma | N/A | RH | GAS | IIA2 |
| 4 | 37 | mixed/bulge | 3 | AGC | AIS | UEA | RT | UEA | IB1 |
| 5 | 50 | mixed/bulge | 3 | adenocarcinoma | adenocarcinoma | N/A | RH | GAS | IB1 |
| 6 | 32 | N/A | N/A | ASC-H | CIN3 | SCC | RT | SCC | IB1 |
| 7 | 34 | solid and cystic | 4 | NILM | No malignancy | N/A | SH | GAS | IB1 |
| 8 | 53 | mixed/bulge | 3 | ASC-US | N/A | N/A | mRH | GAS | IB2 |
| 9 | 49 | mixed/bulge | 3 | N/A | N/A | N/A | SH | GAS | IB1 |
| 10 | 46 | solid and cystic | 4 | adenocarcinoma | adenocarcinoma | N/A | RH | UEA | IB1 |
| 11 | 42 | mixed/bulge | 3 | AGC | N/A | GAS | RH | GAS | IB2 |
| CIN, cervical intraepithelial neoplasia; AIS, adenocarcinoma in situ; UEA, endocervical adenocarcinoma, usual type; SCC, squamous cell carcinoma; GAS, gastric-type mucinous carcinoma; SH, simple hysterectomy; mRH, modified radical hysterectomy; RH, radical hysterectomy; RT, radical trachelectomy; FIGO, International Federation of Gynecology and Obstetrics; N/A, not available. | | | | | | | | |  |

| **Supple Table4. Summary of 9 patients with premalignant diseases** | | | | |  |  |  |  |
| --- | --- | --- | --- | --- | --- | --- | --- | --- |
| Case  No | **Age** | **MRI  findings** | **MRI type** | **Cytology** | **Cervical biopsy** | **Conization** | **Mode of  surgery** | **Postoperative diagnosis** |
| 1 | 45 | mixed/bulge | 3 | NILM | No malignancy | N/A | mRH | Atypical LEGH |
| 2 | 48 | mixed/diffuse | 3 | NILM | No malignancy | N/A | SH | Atypical LEGH |
| 3 | 63 | mixed/diffuse | 3 | adenocarcinoma | CIN1 | NC | SH | Atypical LEGH |
| 4 | 76 | microcystic/diffuse | 3 | AGC | N/A | N/A | SH | Atypical LEGH |
| 5 | 43 | mixed/bulge | 3 | adenocarcinoma | NED | N/A | RH | Atypical LEGH |
| 6 | 51 | mixed/diffuse | 3 | IV | N/A | N/A | RH | Atypical LEGH |
| 7 | 53 | mixed/cosmos | 2 | AGC | LEGH | N/A | SH | Atypical LEGH |
| 8 | 61 | macrocystic | 1 | II | N/A | N/A | SH | LEGH+AIS |
| 9 | 54 | mixed/diffuse | 3 | AGC | AIS | AIS | SH | LEGH+AIS |
| LEGH, lobular endocervical glandular hyperplasia; AIS, adenocarcinoma in situ; SH, simple hysterectomy; mRH, modified radical hysterectomy; RH, radical hysterectomy; N/A, not available. | | | | | | | | |

| **Supple Table 5. Frequencies of MRI findings** | | |  |  |  |  |
| --- | --- | --- | --- | --- | --- | --- |
| Solid and cystic component patterns | |  |  |  |  |  |
|  | Macrocystic | Microcystic | Mixed | Solid and cystic |  |  |
| LEGH | 0 | 2 | 38 | 0 |  |  |
| Malignancy | 0 | 0 | 6 | 4 |  |  |
| Precursors | 1 | 1 | 7 | 0 |  |  |
| NC, TC, etc. | 22 | 2 | 35 | 0 |  |  |
| Total | 23 (19.5%) | 5 (4.2%) | 86 (72.9%) | 4 (3.4%) |  |  |
| P value |  |  |  | <0.0001 |  |  |
|  |  |  |  |  |  |  |
| Tumor location |  |  |  |  |  |  |
|  | entire | lower | middle | upper |  |  |
| LEGH | 15 | 0 | 0 | 25 |  |  |
| Malignancy | 4 | 1 | 1 | 4 |  |  |
| Precursors | 5 | 0 | 0 | 4 |  |  |
| NC, TC, etc. | 20 | 11 | 2 | 26 |  |  |
| Total | 44 (37.3%) | 12 (10.2%) | 3 (2.5%) | 59 (50.0%) |  |  |
| P value |  |  |  | 0.0385 |  |  |
|  |  |  |  |  |  |  |
| Type of structure of cystic component categorized as type 1 to 3 | | |  |  |  |  |
|  | Diffuse growth | | Focal mass like bulging | | Cosmos | |
|  | (+) | (-) | (+) | (-) | (+) | (-) |
| LEGH | 10 | 30 | 8 | 32 | 38 | 2 |
| Malignancy | 1 | 5 | 5 | 1 | 6 | 0 |
| Precursors | 4 | 5 | 3 | 6 | 7 | 2 |
| NC, TC, etc. | 0 | 59 | 0 | 59 | 24 | 35 |
| Total | 15 (13.2%) | 99 (86.8%) | 16 (14.0%) | 98 (86.0%) | 75 (65.8%) | 39 (34.2%) |
| P value |  | <0.0001 |  | <0.0001 |  | <0.0001 |
| LEGH, lobular endocervical glandular hyperplasia; NC, nabothian cyst; TC, tunnel cluster. | | | | | | |
